# Functional Capacity Evaluation and Rehabilitation Strategies in Cardiac Amyloidosis: A Comprehensive Review

**DOI:** 10.3390/jcm14197111

**Published:** 2025-10-09

**Authors:** Anthoula Plevritaki, Konstantinos Volaklis, Eleni Nakou, Constantinos Davos, Ioannis Kopidakis, Eleftherios Kallergis, Eirini Savva, Emmanouil Simantirakis, Aneta Aleksova, Maria Marketou

**Affiliations:** 1 School of Medicine, University of Crete, 71500 Giofirakia, Greece; anthiplevritaki@gmail.com (A.P.); ekallergis@med.uoc.gr (E.K.); savvaeirini@hotmail.com (E.S.); simantir@uoc.gr (E.S.); maryemarke@yahoo.gr (M.M.); 2 Department of Physical Education and Sport Science, Democritus University of Thrace, 69100 Komotini, Greece; volaklis@sport.med.tum.de; 3 School of Medicine, City St George’s University of London, London SW17 0RE, UK; enakou@sgul.ac.uk; 4 Cardiovascular Laboratory, Biomedical Research Foundation Academy of Athens, 11527 Athens, Greece; cdavos@bioacademy.gr; 5 Department of Internal Medicine, Venizeleion Gereral Hospital, 71409 Iraklio, Greece; p.ionnou@uoc.gr; 6 Department of Medical, Surgical and Health Sciences, University of Trieste, 34127 Trieste, Italy; 7 Cardiothoracovascular Department, Azienda Sanitaria Universitaria Giuliano Isontina, 34129 Trieste, Italy

**Keywords:** cardiac amyloidosis, functional capacity, cardiopulmonary exercise testing, 6-min walk test, frailty, rehabilitation, quality of life, transthyretin amyloidosis

## Abstract

Cardiac amyloidosis (CA) is an increasingly recognized cause of restrictive cardiomyopathy characterized by amyloid fibril deposition in the heart, leading to severe functional impairments and poor prognosis. This review aims to provide a comprehensive overview of the pathophysiology of CA, emphasizing the mechanisms underlying functional capacity limitations and highlighting the importance of precise physiological assessment tools. We focus on objective measures such as cardiopulmonary exercise testing, field-based functional tests, and frailty evaluations that are vital for prognosis and tailoring patient care. With recent advances in disease-modifying therapies extending survival, maintaining and improving functional status through multidisciplinary rehabilitation emerges as a crucial therapeutic target. Evidence suggests that structured aerobic and resistance training can enhance exercise tolerance, strength, and quality of life in CA patients, although further research is needed to optimize rehabilitation protocols. By integrating clinical, physiological, and rehabilitative insights, this review underscores the value of a patient-centered approach aimed at preserving functional capacity and improving outcomes in this complex and systemic disease.

## 1. Introduction

Cardiac amyloidosis (CA) represents an increasingly recognized cause of restrictive cardiomyopathy and heart failure, resulting from the extracellular deposition of misfolded amyloid fibrils within myocardial tissue. Historically underdiagnosed due to its protean manifestations and overlap with other cardiac conditions, recent advances in imaging, biomarker detection, and disease-modifying therapies have heightened clinical awareness and diagnostic precision [1,2,3]. Among the various forms, transthyretin amyloidosis (ATTR), both hereditary (ATTRv) and wild-type (ATTRwt), along with immunoglobulin light-chain amyloidosis (AL), account for the vast majority of cardiac involvement [1,2,3]. CA was previously considered rare but recent studies suggest it is substantially more prevalent than once thought, potentially challenging its classification as a rare disease [4]. Autopsy studies have found wild-type ATTR deposits in a substantial proportion of octogenarian heart failure patients, highlighting its clinical significance in the elderly [5]. In older adults, it’s often mistaken for undifferentiated HFpEF, low-flow/low-gradient aortic stenosis, or hypertensive heart disease; in younger patients, for hypertrophic cardiomyopathy [6]. While AL amyloidosis affects both sexes and often presents in mid-life, ATTRwt typically affects men over age 60–70, and specific hereditary ATTR mutations (e.g., Val122Ile in individuals of African descent, Val30Met in endemic areas) confer varying geographic prevalence and onset, underscoring the global nature of this disease [6].

Patients experience refractory heart failure symptoms, arrhythmias, and multi-organ dysfunction that severely limit daily activities. A predominant clinical manifestation is exercise intolerance, as these patients have reduced functional capacity and fatigue that impair their quality of life [7]. Recognizing and addressing functional impairment is critical: recent evidence shows that functional metrics like the 6-min walk distance and peak oxygen uptake correlate strongly with prognosis in cardiac amyloidosis [8,9]. As disease-modifying agents (e.g., tafamidis, patisiran, vutisiran, daratumumab) extend survival, maintaining or enhancing functional status has become a crucial therapeutic goal [10]. Thus, beyond merely treating the amyloid disease process, there is a compelling rationale to focus on preserving and improving patients’ functional capacity through rehabilitation. Rehabilitation interventions have the potential to improve exercise tolerance, enhance quality of life, and possibly favorably influence clinical outcomes in this high-risk population. Given the complexity of cardiac amyloidosis and its systemic nature, a comprehensive, multidisciplinary approach is needed [11,12]. This review provides a thorough and timely synthesis of current knowledge on CR in CA. It emphasizes the importance of evaluating and monitoring patients’ functional capacity—an often overlooked but vital aspect of patient care. Moreover, we emphasize the growing and critical role of CR in the management of CA, especially as advances in disease-modifying therapies have prolonged patient survival, elevating the importance of maintaining quality of life and preserving functional capacity. By elucidating evidence-based rehabilitation strategies and outlining future directions for research and clinical practice, the review serves as a foundational resource for clinicians, rehabilitation specialists, and researchers. It encourages a multidisciplinary, patient-centered approach that goes beyond treating the underlying amyloid pathology to optimize functional status and clinical outcomes actively.

## 2. Search Methodology of the Literature

A comprehensive literature search was conducted to identify relevant studies, reviews, and clinical trials on cardiac amyloidosis, functional capacity evaluation, and rehabilitation strategies. The search included electronic databases such as PubMed/MEDLINE, Embase, and Cochrane Library, covering literature published up to June 2025. Key search terms included “cardiac amyloidosis”, “functional capacity”, “cardiac rehabilitation”, “exercise”, “frailty”, and “quality of life”, used alone or in combination with Boolean operators. Inclusion criteria comprised original research articles, reviews, and clinical guidelines published in English, focusing on adult human populations. Exclusion criteria included non-peer-reviewed articles, conference abstracts without full texts, and studies not directly addressing functional assessment or rehabilitation in cardiac amyloidosis. Reference lists of selected articles were also screened for additional relevant publications. This approach aimed to ensure a thorough and up-to-date synthesis of evidence to support the review’s objectives.

## 3. Pathophysiology of Cardiac Amyloidosis

### 3.1. Types of Cardiac Amyloidosis

Cardiac amyloidosis is mainly caused by two amyloidogenic proteins: immunoglobulin light chains (AL amyloidosis) and transthyretin (ATTR amyloidosis). Both types lead to progressive infiltration of the myocardium, resulting in thickened and stiff ventricles along with diastolic dysfunction. In AL amyloidosis, misfolded monoclonal light chains produced by a clonal plasma cell population exert direct cardiotoxic effects in addition to forming fibrillar deposits [13].

ATTR amyloidosis occurs in two forms: ATTRv, caused by mutations in the transthyretin (TTR) gene, and ATTRwt, which is associated with aging. In both, TTR tetramers dissociate and misfold into insoluble fibrils. ATTRwt mainly affects elderly males and typically follows a slowly progressive course [14]. Amyloid deposits increase ventricular wall thickness without dilating the cavity, leading to restrictive filling patterns, symptoms of heart failure with preserved ejection fraction (HFpEF), reduced stroke volume, and exercise intolerance [15].

Amyloid infiltration also affects the autonomic nervous system, causing orthostatic hypotension [16,17], and deposits in the atria and cardiac conduction system increase the risk of arrhythmias and thromboembolic complications [18,19]. Furthermore, amyloid proteins deposit within the vascular bed-including coronary microvasculature and small vessels-leading to vascular stiffening, impaired myocardial perfusion, and contributing to ischemia and further myocardial dysfunction. This vascular involvement amplifies cardiac impairment and functional limitations [20]. Beyond the heart, systemic amyloid deposition affects the peripheral nervous system, kidneys, and musculoskeletal system, worsening overall functional decline and emphasizing the need for a comprehensive understanding of the multisystem burden in cardiac amyloidosis [21,22].

### 3.2. Mechanisms of Functional Limitations

The mechanisms underlying functional limitations in CA are multifactorial and involve a complex interplay of cardiac and vascular infiltration, pulmonary involvement, autonomic dysfunction, and neurologic pathology [14,15,18,21,22] (Table 1).

Myocardial infiltration by amyloid fibrils leads to restrictive cardiomyopathy, characterized by stiff, thickened ventricles that impair diastolic filling and reduce cardiac output [14,26]. Although patients often maintain a preserved ejection fraction at rest, their ability to augment cardiac output during exercise is significantly compromised, resulting in a reduced cardiac reserve [14,26]. This limitation forces an early reliance on anaerobic metabolism during physical activity, which contributes to rapid fatigue and exercise intolerance. Chronotropic incompetence, typical in this condition, arises from intrinsic conduction system disease and autonomic neuropathy, further limiting the heart’s ability to increase rate and output in response to exertion [26,27]. In some cases, left ventricular outflow tract obstruction may develop, mimicking hypertrophic obstructive cardiomyopathy and representing an additional, though less common, cause of exercise limitation. [28] Arrhythmias, such as atrial fibrillation and atrioventricular block, frequently occur and exacerbate the reduction in cardiac output and patient tolerance for physical activity [17,26].

Beyond cardiac dysfunction, patients with cardiac amyloidosis often exhibit pulmonary and ventilatory abnormalities [9,21,26]. Cardiopulmonary exercise testing (CPET) typically shows reduced peak oxygen uptake and ventilatory inefficiency [7,9,22,26]. These patients tend to breathe rapidly and shallowly with a heightened ventilatory drive, evidenced by an elevated VE/VCO_2_ slope. This pattern reflects increased dead-space ventilation rather than primary lung pathology and signifies inefficient gas exchange, further limiting exercise capacity [7,22,26].

Autonomic nervous system involvement is another critical factor contributing to functional impairment. Patients frequently display an impaired heart rate response to exercise and experience orthostatic hypotension due to autonomic failure [15,16,21]. Chronotropic incompetence is particularly prevalent in ATTR and is a major contributor to early-onset fatigue during exertion [21,27]. Blood pressure instability and impaired vasoconstriction during physical activity further restrict patients’ functional capacity, leading to increased symptoms and reduced exercise tolerance [15,21]. Recent longitudinal data further underscore the interplay between myocardial mechanics and vascular function in CA. In a six-month follow-up, Korela et al. [20] demonstrated significant deterioration in global longitudinal strain (GLS) and progressive arterial stiffness, although the correlation between these parameters was modest (r ≈ 0.3). These findings suggest that systolic dysfunction and vascular impairment may evolve partly independently, compounding exercise intolerance and functional decline.

Peripheral neuropathy, common in both AL and ATTRv, causes sensory loss, muscle weakness, and pain, all of which negatively impact walking ability, endurance, and overall physical activity [19,21,29]. Such neuropathic involvement can lead to gait disturbances and balance issues, increasing the risk of falls and further limiting mobility [21,29]. Sarcopenia-the loss of skeletal muscle mass and strength—and frailty are frequently observed, especially in elderly patients with ATTRwt, compounding physical limitations and impairing performance of daily activities [12].

Additional systemic factors exacerbate functional decline. Renal dysfunction and anemia diminish oxygen delivery to peripheral tissues, intensifying fatigue and reducing endurance [21,23,29]. Gastrointestinal autonomic dysfunction, including symptoms such as malabsorption and altered motility, contributes to nutritional deficiencies, muscle wasting, and frailty, thereby further reducing functional capacity [12,21]. Collectively, these cardiac, pulmonary, autonomic, neurologic, and systemic abnormalities create a profound and multifaceted burden on patients with CA, underscoring the need for comprehensive, multidisciplinary management approaches focused not only on treating the underlying disease but also on optimizing functional status and quality of life [2,12,29]. Cardiac involvement portends serious morbidity and mortality. Historically, untreated AL cardiac amyloidosis had a median survival of only ~6–12 months due to its rapid progression [30]. ATTR amyloidosis follows a more indolent course, with survival measured in years, but still leads to a fatal outcome if untreated [31]. In ATTR, tafamidis improves survival but morbidity remains substantial [32].

## 4. Physiological Assessment in Cardiac Amyloidosis

Older, multimorbid patients with CA benefit from comprehensive assessment of physical and cognitive impairments to optimize management and prognosis [12,33]. Functional status in geriatrics reflects ability perform Activities of Daily Lives (ADLs)/Instrumental Activities of Daily Living (IADLs); standard questionnaires are informative but self-reported and less sensitive to preclinical decline [34,35]. Although these tools offer valuable insight, they may not detect subtle or early functional deterioration, particularly in frail phenotypes [36]. Merging subjective and objective measures provides a more thorough understanding of a patient’s actual functional condition and aids in developing tailored management strategies [33,37] (Table 2).

### 4.1. Objective Measures

**Figure 1:**
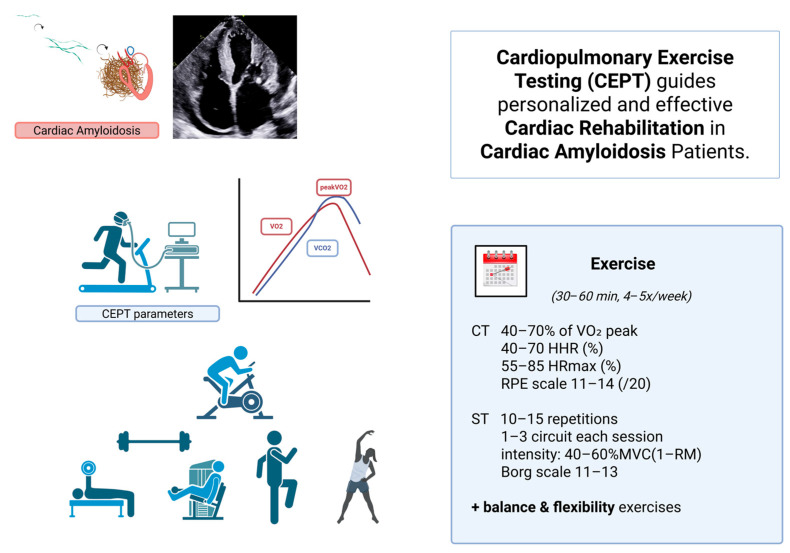
Cardiopulmonary Exercise Testing (CEPT)–guided cardiac rehabilitation in patients with cardiac amyloidosis. Misfolding of monoclonal light chains or dissociated transthyretin (TTR) tetramers triggers amyloid fibrillogenesis. Misfolded monomers aggregate into oligomers, which further organize into insoluble amyloid fibrils that accumulate in the extracellular matrix of various organs—particularly the heart—resulting in cardiac amyloidosis. Cardiopulmonary exercise testing (CEPT) provides individualized parameters (e.g., VO_2_ peak, ventilatory thresholds) that guide safe and effective exercise prescription. A structured rehabilitation program includes aerobic (CT) and strength training (ST) components performed 4–5 times per week for 30–60 min, with intensity tailored to 40–70% of VO_2_ peak, 55–85% of HRmax, or RPE 11–14. Strength training involves 10–15 repetitions per exercise, 1–3 circuits at 40–60% of maximal voluntary contraction (1-RM), supplemented with balance and flexibility exercises. This personalized approach optimizes functional recovery and quality of life in patients with cardiac amyloidosis. (VO_2_ peak: peak oxygen uptake, HRR: heart rate reserve, HRmax (%): heart rate max, RPE scale: ratings of perceived exertion, CT: continuous training, ST: strength training, MVC: maximal voluntary contraction, 1-RM: one-repetition maximum, HIT, high-intensity interval training, IT: interval training, LIT: low-intensity interval training, RST: resistance strength training.

Symptom-limited CPET with gas exchange is the gold standard to quantify functional capacity and delineate mechanisms (cardiac, pulmonary, peripheral) [38]. Beyond peak maximum rate of oxygen consumption (peak VO_2_) and the ratio of minute ventilation (VE) to carbon dioxide production (VCO_2_) that assesses ventilatory efficiency, VE/VCO_2_ slope independently predicts clinical outcome across CA phenotypes [22,26,38] and may tailor the prescription of CR programs (Figure 1). Peak VO_2_ is a critical parameter for functional assessment and risk stratification in cardiac amyloidosis. Studies suggest that a peak VO_2_ below 12–14 mL/kg/min is associated with worse prognosis, including higher rates of hospitalization and mortality [22,26]. These thresholds can guide clinical decisions such as tailoring rehabilitation intensity and considering advanced therapies including transplantation. Moreover, a 6MWT distance of <350 m was associated with a 2.2-fold higher risk of mortality [39].

In addition, 6-min walk test (6MWT) and 5-min walk (gait speed) are practical objective measures [8,41]. In ATTR-CA, both a lower baseline six-minute walk distance (6MWT) and a greater decline in 6MWT over one year are independently associated with increased all-cause and cardiovascular mortality. When combined with circulating biomarkers, changes in 6MWT can further refine risk stratification and improve prognostic accuracy [8,31,33].

The Short Physical Performance Battery (balance, gait speed, chair rises; score 0–12) may predict disability and adverse cardiovascular outcomes [41,42]. In ATTR-CA, SPPB independently associates with mortality, underscoring the prognostic value of lower-extremity function [33].

### 4.2. Frailty Status Assessment

Frailty- a syndrome of reduced physiologic reserve—predicts falls, hospitalization, and mortality; prevalence in CA is high (≈33–57%) and exceeds that of community-dwelling older adults [46,47,48,49]. Shared mechanisms (inflammation, oxidative stress) support a bidirectional relationship between frailty and amyloidosis [12,14].

Phenotypic frailty: Fried’s criteria (weight loss, exhaustion, low activity, slow gait <0.8 m/s, weakness by grip strength) classify non-frail, pre-frail, and frail [50].

Global frailty: The Clinical Frailty Scale (1–9) captures overall reserve; ≥5 indicates frailty [51].

Frailty relates to quality of life and disease severity in CA [33,36], and independently predicts mortality across ages, genotypes, and stages; adding frailty to biomarkers (e.g., N-terminal pro-B-type natriuretic peptide) improves risk stratification [46,48].

### 4.3. Quality of Life and Mood Disorders

CA’s burdens (dyspnea, fatigue, weakness) substantially impair health-related quality of life and mood [52]. Generic (SF-36, Hospital Anxiety and Depression Scale) and heart failure specific instruments (Kansas City Cardiomyopathy Questionnaire, Minesota Living with Heart Failure Questionnaire) are used in CA [53,54]. Neuropathy burden can be tracked with PNDS or Norfolk QoL-DN [55]. A validated disease-specific instrument-the ATTR-QOL—captures both cardiomyopathy and polyneuropathy manifestations across ATTRv/ATTRwt [56]. Anxiety/depression are common; ~half of patients may screen positive, supporting routine mental-health assessment and support [57]. Health-related quality of life impairment tracks with disease severity and outcome [58].

## 5. Cardiac Rehabilitation Programs in the Management of Cardiac Amyloidosis

Cardiorespiratory response to exercise significantly worsened over a short period of time in patients with ATTR-CM. Serial CPET may be useful to identify early disease progression. CR is essential in the management of CA addressing various factors that contribute to functional decline, such as cardiac restrictions, neuromuscular issues, and skeletal muscle adaptations [15]. Given the restrictive nature of cardiomyopathy and the systemic involvement typical of CA, a thorough interdisciplinary rehabilitation approach is crucial for maintaining functional capacity, improving quality of life, and possibly enhancing clinical outcomes. Although evidence in CA is not extensive, parallels can be drawn from research in similar conditions like hypertrophic cardiomyopathy and heart failure with preserved ejection fraction, where structured exercise regimens have shown benefits in exercise tolerance, symptoms, and overall well-being [59,60,61]. These results indicate that appropriately selected CA patients might also gain from customized exercise programs. Initial studies in CA indicate that structured aerobic training is both feasible and safe, with the most significant improvements seen in patients with lower biomarker levels and better initial functional status [62]. The ongoing ERICA study will randomize ATTRwt-CA patients into a control group and a training group [61]. Primary endpoint will be the distance obtained at the 6 min walk test. Quality-of-life, peak oxygen consumption, left and right heart architecture and function, and natriuretic peptides will be secondary endpoint. Notably, the trial will assess a holistic rehabilitation program that combines moderate-intensity aerobic exercise (~40–50% peak VO_2_) with nutritional advice, psychological support, and assistance with smoking cessation [63]. This multidisciplinary approach seeks to enhance peak oxygen uptake, left ventricular function, biomarker profiles, and health-related quality of life, thereby providing essential evidence to inform clinical practice.

Resistance training also plays a vital role, especially since sarcopenia and muscle loss are frequently observed in CA patients—particularly older individuals and those with chronic heart failure—leading to difficulties in performing ADLs and reduced health-related quality of life [6]. Effective resistance training programs focus on rhythmic, dynamic movements executed at low to moderate intensity (around 40–60% of one-repetition maximum), with brief work intervals (no longer than 45 s) and ample rest periods (at least a 1:2 work-to-rest ratio) to minimize stress on the heart [62,63,64,65,66]. Alternative strategies include using lightweight free weights, elastic resistance bands, or segment-specific training routines adapted for patients with limited capacity. In addition, some other studies are exploring the benefits of CR in CA.

The following small-scale studies are among the few that specifically focus on CA within the broader context of CR research. These programs are assessing the impact of supervised exercise on functional capacity, muscle strength, and health-related quality of life, with early data suggesting good adherence and safety [63,64]. Researchers are also examining optimal exercise intensity, duration, and modalities to maximize benefits while minimizing risks in this vulnerable population [64]. A pilot study combining aerobic and strength training in patients with ATTR cardiomyopathy receiving tafamidis reported high adherence rates and significant gains in peak oxygen uptake and health-related quality of life over a 16-week timeframe [64]. Ongoing studies aim to build a robust evidence base to guide personalized rehabilitation strategies and improve clinical outcomes in cardiac amyloidosis [65,66]. Although randomized controlled trials on cardiac rehabilitation in cardiac amyloidosis are limited, strong clinical rationale supports its use (Table 3).

The severe functional impairment from myocardial infiltration, autonomic dysfunction, and muscle wasting suggests that tailored rehabilitation can improve exercise capacity, reduce frailty, and enhance quality of life. Evidence from related cardiac conditions indicates structured exercise is beneficial and applying similar principles-combining moderate aerobic and resistance training with nutritional and psychosocial support-can optimize outcomes in CA patients.

### Rehabilitation Protocols, Safety Considerations, and Patient Selection in CA

Rehabilitation protocols for patients with CA should be individualized and carefully supervised, given the complex multisystem involvement and potential cardiovascular risks inherent to this population. Current evidence from similar populations, though limited, supports the use of combined moderate-intensity aerobic and resistance training tailored to each patient’s functional status and disease severity [59,60]. Aerobic exercise sessions are typically prescribed at 40–70% of peak oxygen uptake (VO_2_ peak) as determined by CPET, with a frequency of 3–5 times per week and session durations ranging from 30 to 60 min. Resistance training should focus on major muscle groups, performed with low to moderate intensity (e.g., 40–60% of one-repetition maximum), emphasizing dynamic, rhythmic movements with appropriate rest intervals tailored to each patient. The intensity and the duration of the program can be gradually increased every 2–4 weeks. Incorporating balance and flexibility exercises can address neurological and musculoskeletal complications such as peripheral neuropathy and sarcopenia frequently observed in CA patients.

A key limitation of this review is the current scarcity of high-quality, disease-specific evidence supporting rehabilitation interventions in CA. Most recommendations are necessarily extrapolated from related cardiomyopathies and heart failure syndromes, reflecting the paucity of randomized controlled trials and guideline-based graded recommendations specifically for this population. This limitation underscores the pressing need for further rigorous research to establish evidence-based rehabilitation protocols tailored to the unique pathophysiology and multisystem involvement of cardiac amyloidosis.

Safety monitoring is paramount during rehabilitation. Clinicians should closely monitor for arrhythmias, chronotropic incompetence, blood pressure instability, and symptom exacerbation during exercise sessions. A multidisciplinary team approach involving cardiologists, physiotherapists, and neurologists is advisable to optimize patient management and promptly address complications.

Exercise prescription should consider baseline functional capacity (e.g., 6 min walk test distance, frailty indices, NYHA class, risk of falls and cardiac biomarker levels. Comorbidities such as autonomic dysfunction, significant conduction abnormalities, or orthopedic limitations require personalized adjustments in exercise prescription and closer supervision. Ongoing studies such as the ERICA trial [61] are expected to provide higher-level evidence to refine rehabilitation protocols further and define optimal exercise modalities, intensity, and safety parameters specifically for the CA population.

Finally, CA includes diverse subtypes—primarily AL and ATTR (hereditary and wild-type)—with distinct clinical courses influencing functional capacity and rehabilitation potential. However, there is a lack of strong evidence supporting rehabilitation interventions specifically in advanced disease stages, such as severe heart failure (NYHA class IV) or extensive multisystem involvement. Consequently, rehabilitation in these patients requires cautious individualized decision-making, thorough multidisciplinary evaluation, and realistic expectations regarding achievable outcomes.

## 6. Future Perspectives

Future research should focus on developing evidence-based rehabilitation protocols tailored to the specific pathophysiological mechanisms and multisystem involvement typical of cardiac amyloidosis. High-quality, randomized controlled trials are necessary to determine the optimal exercise modalities, intensity, duration, and progression for different CA phenotypes and disease stages. Dedicated safety monitoring standards and multidisciplinary team involvement—including cardiologists, neurologists, physiotherapists, and nutritionists—should become integral parts of rehabilitation programs. Expanding rehabilitation access, integrating digital health solutions, and developing personalized exercise prescriptions based on objective physiological assessments will likely advance outcomes for these high-risk patients. Ultimately, incorporating CR into routine CA care may yield substantial benefits for patient-centered outcomes, quality of life, and long-term prognosis, but robust clinical evidence is needed to establish best practices for this complex population.

## 7. Conclusions

Incorporating focused CR programs into standard care for CA presents considerable potential for reducing functional decline and improving patient-centered outcomes. Future research should aim to develop evidence-based guidelines that optimize the safety, effectiveness, and accessibility of rehabilitation strategies tailored to this complex and high-risk group of patients. Ultimately, this review emphasizes that rehabilitation is not merely supportive care but a potentially transformative element in the comprehensive management of cardiac amyloidosis, with broader applications across cardiovascular diseases. Its value lies in advancing awareness, guiding best practices, and inspiring further investigation into rehabilitation’s role in improving prognosis and quality of life among CA patients. Clinicians should consider individualized, carefully monitored programs while ongoing research works to establish evidence-based guidelines for this population. However, evidence is urgently required to identify the best exercise modalities, intensity, duration, and progression specifically for the CA population.

## Figures and Tables

**Table 1:** Summary of Organ System Involvement and Functional Impact in Systemic Amyloidosis.

Organ/System	Pathological Changes in Amyloidosis	Contribution to Physical Intolerance
Heart	Restrictive cardiomyopathy, heart failure, arrhythmias [1,2,3,4,9,12,13,14,17]	Dyspnea, edema, reduced exercise tolerance, fatigue
Kidneys	Proteinuria, nephrotic syndrome, renal failure [13,21,23]	Fatigue, fluid overload, electrolyte imbalance
Peripheral nerves/Autonomic nervous system	Amyloid neuropathy, dysautonomia [13,16,17,21,24]	Muscle weakness, sensory loss, orthostatic hypotension, blood pressure instability, exercise intolerance
Gastrointestinal tract	Malabsorption, motility disorders, bleeding [13,19,21]	Weight loss, malnutrition, diarrhea, fatigue
Liver	Hepatomegaly, cholestasis [13,19,21]	Abdominal discomfort, early satiety, decreased metabolism
Lungs	Interstitial deposition, pleural effusion [19,21]	Reduced exercise capacity, hypoxemia, dyspnea
Musculoskeletal system	Muscle infiltration, joint involvement, neuropathy, amyloid infiltration of tendons [13,19,21]	Weakness, stiffness, reduced mobility, tendon rupture (Popeye sign in biceps tendon rupture) [25]
Skin	Purpura, macroglossia, waxy thickening [13,19,21]	Pain, functional limitations (speech, swallowing)
Spleen	Splenomegaly, hypersplenism [19,21]	Cytopenias, increased fatigue, infection risk
Hematologic	Plasma cell dyscrasia (in AL amyloidosis) [13,19,21]	Anemia-related fatigue, reduced oxygen delivery

**Table 2:** Common tools for the assessment of physical functions and psychological well-being among patients with cardiac amyloidosis reported in the literature.

Domain	Assessment Tools
Functional capacity	Cardiopulmonary exercise testing [6,9,22,37,38]; 6-min walk test [8,39,40]; Short Physical Performance Battery (balance, walking speed, sit-to-stand chair test) [41,42]
Frailty criteria	Weight loss; handgrip strength test; gait speed; low physical activity [12,33,36]
Nutritional status	Geriatric Nutritional Risk Index; Dietitian support [33]
Cognitive performance	Mini Mental State Examination [33]
Anxiety and depression	Major Depression Inventory; Geriatric Depression Scale; Hospital Anxiety and Depression Scale (HADS) [43]
Quality of life	Kansas City Cardiomyopathy Questionnaire (KCCQ) [44]; Minnesota Living with Heart Failure Questionnaire (MLHFQ) [45]

**Table 3:** Existing and ongoing trials on the impact of treatment on functional capacity parameters in cardiac amyloidosis.

Study/Trial Name	Condition/Population	Intervention/Treatment Type	Key CPET Outcomes Measured	Status/Notes
Impact of Tafamidis and Optimal Treatment on Physical Performance	Transthyretin amyloid cardiomyopathy	Tafamidis with background therapy	Peak VO_2_, VE/VCO_2_ slope	Completed; significant improvements in less advanced disease [32].
Serial Changes in CPET Parameters	Transthyretin cardiac amyloidosis	Observational (untreated)	Peak VO_2_, VE/VCO_2_ slope, exercise time, tolerance	Completed; untreated patients showed worsening CPET measures [37].
ERICA (Exercise Training and Rehabilitation in Cardiac Amyloidosis) (NCT06412432)	Wild-type ATTR-CA	Tailored supervised exercise training	6MWT, peak VO_2_, QoL, echocardiography, natriuretic peptides	Invitation-only; investigates tailored rehab program in ATTRwt-CA [59].
Exercise Training in Transthyretin Cardiac Amyloidosis (NCT05797857)	Transthyretin cardiac amyloidosis	Personalized exercise training	Peak VO_2_, 6MWT, VE/VCO_2_ slope	Recruiting; aims to develop a personalized exercise program to improve functional capacity [65].
CAPACITY (Cardiac Amyloidosis and Physical Activity) (NCT06096675)	Cardiac amyloidosis	Supervised cardiac rehabilitation	Peak VO_2_, VE/VCO_2_ slope, 6MWT, QoL (KCCQ), chronotropic incompetence	Recruiting; 12-week supervised rehab program [66].

## Data Availability

Not applicable.

## Abbreviations

The following abbreviations are used in this manuscript.

ADLs	Activities of Daily Lives
AL amyloidosis	Light-chain amyloidosis
ATTR	Transthyretin amyloidosis
ATTRv ATTRwt	Hereditary transthyretin amyloidosis Wild type transthyretin amyloidosis
CA CR	Cardiac Amyloidosis Cardiac Rehabilitation
CPET	Cardiopulmonary exercise test
IADLs	Instrumental Activities of Daily Living
6MWT	6 min walk test
